# Serratia marcescens Bloodstream Infections in a Neonatal Intensive Care Unit: A Retrospective Case Series From Rabat, Morocco

**DOI:** 10.7759/cureus.114991

**Published:** 2026-08-22

**Authors:** Chaymaa El Messari, Salah Saghir, Mohammed Sellouti, Anas Ayad, Rachid Abilkassem

**Affiliations:** 1 Neonatal Medicine and Intensive Care Unit, Mohammed V Military Teaching Hospital, Rabat, MAR

**Keywords:** infection control, neonatal sepsis, prematurity, serratia marcescens, umbilical catheter

## Abstract

Background

*Serratia marcescens* is an opportunistic Gram-negative bacillus increasingly implicated in neonatal intensive care unit (NICU) infections and severe neonatal sepsis, particularly among preterm infants.

Methods

We conducted a retrospective observational case series in the NICU of Mohammed V Military Teaching Hospital, Rabat, Morocco, between August 2024 and August 2025. Neonates with at least one positive blood culture for *S. marcescens* associated with clinical signs of sepsis were included. Clinical, laboratory, microbiological, imaging, therapeutic, and outcome data were analyzed.

Results

Sixteen neonates were identified; 12/16 (75.0%) were born before 34 weeks of gestation, and 9/16 (56.3%) had a birth weight below 1,500 g. Respiratory distress and hemodynamic instability were the predominant clinical manifestations. Thrombocytopenia (platelet count <50,000/µL) was observed in 6/16 (37.5%) neonates, and elevated C-reactive protein (CRP) levels were frequent. Cranial ultrasonography was normal in most patients; grade I intraventricular hemorrhage was identified in 5/16 (31.3%). One neonate developed multiple contrast-enhancing brain lesions on magnetic resonance imaging (MRI), consistent with multifocal brain abscess formation. All patients received empirical broad-spectrum antibiotic therapy, subsequently adjusted according to antimicrobial susceptibility testing. Overall mortality was 7/16 (43.8%).

Conclusion

This single-center case series demonstrates the potential severity of *S. marcescens* bloodstream infection in hospitalized neonates. The high observed mortality highlights the importance of timely microbiological diagnosis, susceptibility-guided treatment, and rigorous infection-prevention surveillance. Molecular typing and structured outbreak investigations are warranted when temporal clustering is suspected.

## Introduction

Neonatal sepsis remains a major cause of morbidity and mortality in neonatal intensive care units (NICUs) [1]. Data on neonatal *Serratia marcescens* infection from Morocco and North Africa remain scarce, and most published series originate from European, Asian, or Latin American NICUs; this report contributes region-specific clinical, microbiological, and outcome data that may inform local infection-control and empirical-therapy practices. Although early-onset neonatal sepsis is predominantly caused by organisms such as *Escherichia coli* and group B *Streptococcus*, and late-onset, healthcare-associated sepsis is more frequently attributed to *Klebsiella* spp., *Enterobacter* spp., *Pseudomonas* spp., and coagulase-negative staphylococci, *S. marcescens* has increasingly emerged as a significant late-onset, healthcare-associated pathogen in vulnerable neonates [1-3]. Severe invasive bacterial infections in neonates may initially present with nonspecific manifestations, including respiratory distress and feeding difficulties, and may progress rapidly, emphasizing the importance of considering the timing of disease onset in clinical assessment [4]. *S. marcescens* is capable of surviving in moist hospital environments and can colonize medical equipment, solutions, and healthcare workers’ hands, facilitating cross-transmission and healthcare-associated clusters [2].

In this report, “high-risk neonates” refers to preterm and/or low-birth-weight infants requiring invasive devices (umbilical catheters and mechanical ventilation) and prolonged NICU stays. Gestational age categories were defined as follows: extremely preterm (<28 weeks), very preterm (28-<32 weeks), and moderate-to-late preterm (32-<37 weeks); each successively lower gestational-age category is associated with greater immaturity of the immune system, skin barrier, and organ function and, therefore, with higher susceptibility to healthcare-associated infection. Birth weight was categorized as low birth weight (LBW, <2,500 g), very low birth weight (VLBW, <1,500 g), and extremely low birth weight (ELBW, <1,000 g), consistent with World Health Organization definitions.

We distinguish colonization (organism recovery without clinical signs) from culture-confirmed infection (positive blood culture with clinical signs of sepsis, as defined below), healthcare-associated infection (infection occurring after a defined interval following admission), temporal clustering (multiple cases occurring within a defined period without confirmed epidemiological linkage), and a confirmed outbreak (temporal clustering supported by molecular or epidemiological evidence of transmission). In neonates, major risk factors include prematurity, LBW, prolonged hospitalization, exposure to broad-spectrum antibiotics, and invasive procedures, particularly indwelling catheters and mechanical ventilation [1,2,5]. The clinical presentation is often nonspecific, which may delay diagnosis and appropriate management [1,2]. In addition, intrinsic and acquired antimicrobial resistance mechanisms, including chromosomal AmpC β-lactamase production, may complicate treatment strategies in some isolates [2,6]. Severe complications such as meningitis and brain abscesses have also been reported [1,2].

Cases occurred throughout the one-year study period rather than within a discrete time window; a possible healthcare-associated source was considered, although molecular confirmation was unavailable. The objective was to describe the demographic, clinical, laboratory, microbiological, radiological, therapeutic, and outcome characteristics of neonates with culture-confirmed *S. marcescens* sepsis admitted to our NICU and to summarize the infection-control implications of the observed cases.

## Materials and methods

Study design and setting

This retrospective observational case series was conducted in the level III NICU of Mohammed V Military Teaching Hospital, a national military referral hospital in Rabat, Morocco, admitting infants of military-affiliated families. The unit comprises eight beds for intensive/critical care and six beds for lower-acuity care, with a nurse-to-patient ratio of approximately 1:3-4, and a typical annual admission volume of approximately 400 neonates. The study was conducted between August 10, 2024, and August 24, 2025. During this period, 377 unique neonates were admitted to the NICU, consistent with the unit’s typical annual volume. Sixteen of these neonates (16 unique infants, each with a single infection episode) met the inclusion criteria for culture-confirmed *S. marcescens* sepsis, corresponding to 4.2% of admissions. This proportion should not be interpreted as an incidence density, as patient-day or central-line-day denominators were not available. This study was designed as a retrospective case series rather than a prospective outbreak investigation; no environmental or molecular investigation was performed at the time of case occurrence. Our unit has not experienced other formally documented microbial outbreaks during the period surrounding this study; however, systematic outbreak surveillance was not in place, so this cannot be fully quantified retrospectively.

Case definition

Neonates were included if they met both of the following criteria: (i) at least one positive blood culture for *S. marcescens*, and (ii) at least one clinical sign of sepsis, defined as hemodynamic instability (hypotension, prolonged capillary refill time, and mottled skin), respiratory distress or apnea, temperature instability, lethargy or feeding difficulty, or sclerema, in association with elevated C-reactive protein (CRP) (>10 mg/L). In six patients, umbilical catheter-tip cultures were also positive for *S. marcescens*.

Healthcare-associated (nosocomial) infection was operationally defined as culture-confirmed *S. marcescens* sepsis occurring more than 48 hours after NICU admission. All 16 cases met this definition; the hospital day at infection onset ranged from day 3 to day 66. None represented vertically acquired or early-onset (<72 hours) infection. One neonate (Case 15) was admitted directly at 28 days of life, representing a clearly late-onset, healthcare-associated presentation.

Repeat blood cultures were obtained approximately every 48 hours after the initial positive culture and were continued at 48-hour intervals if the culture remained positive or if no clinical improvement was observed, until microbiological clearance was documented.

Inclusion and exclusion criteria

Neonates admitted to the NICU during the study period were eligible if they had at least one positive blood culture for *S. marcescens* associated with clinical signs of sepsis, as defined above.

Neonates were excluded if they had bloodstream infections caused by microorganisms other than *S. marcescens*, polymicrobial bloodstream infections, major congenital malformations or congenital heart disease that could significantly influence clinical outcomes, or incomplete medical records that precluded adequate data collection. Neonates with major congenital malformations or congenital heart disease were excluded because mortality in this subgroup is frequently attributable to the underlying structural condition itself, independent of infection; their inclusion would have precluded reliable attribution of death to *S. marcescens* infection specifically, introducing a major confounder for the primary outcome. We acknowledge that this exclusion may nonetheless introduce selection bias by removing a high-risk subgroup.

Data collection

For each patient, the following data were collected: demographic characteristics (sex, gestational age, birth weight, and inborn/outborn status), clinical presentation (hemodynamic status, respiratory distress, and septic appearance), use of invasive devices (umbilical venous catheter (UVC) and mechanical ventilation), laboratory findings (complete blood count, CRP, and microbiological cultures), imaging findings (cranial ultrasonography and magnetic resonance imaging (MRI) when available), antibiotic therapy, length of hospital stay, and outcome (survival or death). Procalcitonin levels were not routinely measured in our unit during the study period and were therefore unavailable for analysis. Length of hospital stay ranged from 12 to 90 days. Fifteen of 16 neonates (93.8%) were inborn; one neonate (Case 15) was outborn. Several neonates received empirical antibiotic therapy at birth because of prematurity or initial suspicion of infection, without bacteriological confirmation of a distinct preceding infection. No neonate had a history of prior surgery or exposure to invasive devices other than the UVC and respiratory support. Per unit protocol, blood cultures were obtained at the onset of hemodynamic instability, with simultaneous initiation of empirical antibiotic therapy pending culture results (time to active treatment: day 0). Respiratory support was initiated because of the infection episode in all cases, rather than preceding it. Lumbar puncture was not performed in this cohort, as patients were hemodynamically unstable and had isolated thrombocytopenia at presentation, both of which precluded the procedure. Post-discharge follow-up duration was not available. The primary outcome was all-cause in-hospital mortality.

Thrombocytopenia was defined as a platelet count <150,000/µL, and severe thrombocytopenia as <50,000/µL. “Septic appearance” was defined as the clinical gestalt of an unwell, poorly perfused infant, encompassing pallor or mottled skin, lethargy, and temperature instability, as assessed by the attending neonatologist.

Statistical analysis

Given the small sample size and the absence of an uninfected comparator group, no inferential statistical analysis of risk factors was performed. Continuous variables were summarized using the median and range, while categorical variables were presented as frequencies and percentages.

Microbiological identification was performed using standard automated laboratory methods available in our institution. Antimicrobial susceptibility testing was interpreted according to the Clinical and Laboratory Standards Institute (CLSI) M100 document, 34th edition (2024), with minimum inhibitory concentrations (MICs) determined for all tested agents. The specific automated identification/susceptibility system (manufacturer and model) was not retrospectively documented in the available laboratory records and could not be confirmed. No phenotypic or molecular testing for extended-spectrum β-lactamases, carbapenemases, or aminoglycoside-resistance mechanisms was performed.

All *S. marcescens* isolates recovered during the study period exhibited an identical antimicrobial susceptibility profile. The isolates were resistant to ampicillin (MIC >8 mg/L), amoxicillin-clavulanate (MIC >8 mg/L), cefoxitin (MIC 32 mg/L), cefadroxil (MIC >16 mg/L), amikacin (MIC 32 mg/L), and tobramycin (MIC 8 mg/L), while remaining susceptible to ticarcillin (MIC 4 mg/L), piperacillin-tazobactam (MIC 2 mg/L), imipenem (MIC 0.5 mg/L), cefotaxime (MIC 0.5 mg/L), gentamicin (MIC 1 mg/L), trimethoprim-sulfamethoxazole (MIC 0.0625 mg/L), ciprofloxacin (MIC <0.06 mg/L), and levofloxacin (MIC 0.125 mg/L). Fosfomycin (MIC ≤32 mg/L) was reported by the laboratory as susceptible; however, no CLSI interpretive breakpoint is validated for *S. marcescens* or for bloodstream infection (CLSI fosfomycin breakpoints are validated only for *E. coli* in uncomplicated urinary tract infection), and this result should therefore be interpreted with caution. Cefepime was not tested (Table 1).

**Table 1:** Antimicrobial Susceptibility Profile of Serratia marcescens Isolates (n = 16) *Cefepime (fourth-generation cephalosporin) was not tested; no claim of susceptibility to this class can be made. †No CLSI interpretive breakpoint is validated for *Serratia marcescens* or for bloodstream infection; CLSI fosfomycin breakpoints are validated only for *Escherichia coli* in uncomplicated urinary tract infection. This result should be interpreted with caution. CLSI: Clinical and Laboratory Standards Institute; MIC: minimum inhibitory concentration

Antibiotic class	Antibiotic	Result	MIC (mg/L)
Penicillins	Ampicillin	Resistant	>8
	Amoxicillin-clavulanate	Resistant	>8
	Ticarcillin	Susceptible	4
	Piperacillin-tazobactam	Susceptible	2
Carbapenems	Imipenem	Susceptible	0.5
Cephalosporins	Cefoxitin	Resistant	32
	Cefadroxil (1st gen)	Resistant	>16
	Cefotaxime (3rd gen)*	Susceptible	0.5
Aminoglycosides	Gentamicin	Susceptible	1
	Tobramycin	Resistant	8
	Amikacin (30 µg)	Resistant	32
Sulfonamides	Trimethoprim-sulfamethoxazole	Susceptible	0.0625
Fluoroquinolones	Ciprofloxacin (5 µg)	Susceptible	<0.06
	Levofloxacin	Susceptible	0.125
Other agents	Fosfomycin†	Susceptible	≤32

Ethical considerations

The study was approved by the Comité d’Éthique pour la Recherche Biomédicale de l’Hôpital Militaire d’Instruction Mohammed V under approval number 1985, dated 12/10/2025. Given the retrospective design and the use of anonymized clinical data, the requirement for individual parental informed consent was waived by the ethics committee. Patient data were anonymized prior to analysis.

## Results

During the study period, 377 neonates were admitted. Sixteen unique neonates met the prespecified definition of culture-confirmed *S. marcescens* sepsis, corresponding to 4.2% of admissions. Of these, nine were male and seven were female. Twelve neonates (75.0%) were born before 34 weeks of gestation, and nine (56.3%) had a birth weight below 1,500 g. Gestational age ranged from 31 to 39 weeks (median, 33 weeks), and birth weight ranged from 1,100 g to 3,100 g (median, 1,470 g). Apgar scores ranged from 6 to 9 at one minute (median, 9) and from 7 to 10 at five minutes (median, 9.5). Fifteen of 16 neonates (93.8%) had an UVC. Seven neonates (43.8%) died during hospitalization. Fifteen of 16 neonates (93.8%) were inborn; one neonate (Case 15) was outborn and was admitted directly at 28 days of life (Table 2).

**Table 2:** Clinical, Laboratory, and Outcome Characteristics of Neonates with Serratia marcescens Infection (n = 16) *Case 15: admitted directly at 28 days of life (not from birth). †Case 16: experienced a second *Serratia marcescens* bacteremic episode at day 30 of life and was diagnosed with multifocal brain abscesses on MRI at day 60 of life. GA: gestational age; BW: birth weight; UVC: umbilical venous catheter; CRP: C-reactive protein; CSF: cerebrospinal fluid; IVH: intraventricular hemorrhage; CPAP: continuous positive airway pressure; NIV: non-invasive ventilation; LOS: length of stay; MRI: magnetic resonance imaging

Case	Sex	GA (wks)	BW (g)	Apgar (1/5min)	Inborn/Outborn	Hospital Day at Infection	UVC (dwell time)	Catheter-Tip Culture	CSF	Respiratory Support (duration)	Platelets (/µL)	CRP (mg/L)	Cranial Ultrasonography	LOS (days)	Outcome
1	M	33	1,290	_9/10_	Inborn	Day 4	Yes (7d)	Positive	Not performed	CPAP (6d)	5,000	121	Normal	15	Survived
2	F	33	1,600	_8/9_	Inborn	Day 4	Yes (7d)	Positive	Not performed	CPAP (12d)	7,000	90	Normal	12	Died
3	M	32	1,350	_7/8 _	Inborn	Day 7	Yes (8d)	Negative	Not performed	NIV (3d)	7,000	101	Grade I IVH	20	Died
4	M	33	1,750	_8/9_	Inborn	Day 8	Yes (9d)	Positive	Not performed	Mechanical ventilation (9d)	104,000	78	Grade I IVH	17	Died
5	M	33	1,550	_6/7_	Inborn	Day 11	Yes (5d)	Positive	Not performed	CPAP (8d)	77,000	27	Not available	20	Died
6	M	39	3,100	_9/10_	Inborn	Day 9	Yes (8d)	Positive	Not performed	CPAP (5d)	132,000	86	Grade I IVH	30	Survived
7	M	31	1,350	_7/8_	Inborn	Day 40	Yes (9d)	Positive	Not performed	Mechanical ventilation (6d)	77,000	27	Grade I IVH	60	Died
8	F	31	1,250	_9/10_	Inborn	Day 7	Yes (9d)	Negative	Not performed	CPAP (9d)	66,000	113	Normal	28	Died
9	F	36	2,600	_9/10_	Inborn	Day 4	Yes (7d)	Negative	Not performed	CPAP (3d)	77,000	113	Normal	17	Survived
10	F	31	1,150	_9/10_	Inborn	Day 6	Yes (7d)	Negative	Not performed	CPAP (7d)	24,000	50	Normal	13	Died
11	M	34	1,530	_8/9_	Inborn	Day 4	Yes (5d)	Negative	Not performed	CPAP (3d)	29,000	15	Normal	28	Survived
12	F	33	1,490	_7/8_	Inborn	Day 4	Yes (7d)	Negative	Not performed	CPAP (4d)	12,000	63	Grade I IVH	26	Survived
13	F	31	1,450	_6/8_	Inborn	Day 10	Yes (2d)	Negative	Not performed	CPAP (5d)	56,000	160	Normal	38	Survived
14	F	31	1,200	_9/10_	Inborn	Day 4	Yes (5d)	Negative	Not performed	CPAP (6d)	67,000	108	Normal	34	Survived
15*	M	38	2,550	_9/10_	Outborn	Day 3	No	N/A	Not performed	Oxygen therapy (14d)	129,000	33	Normal	14	Survived
16†	M	31	1,100	_9/10_	Inborn	Day 4	Yes (9d)	Negative	Not performed	CPAP (10d)	170,000	4.8	Grade II IVH	90	Survived

Clinical characteristics

Most patients presented with signs of sepsis and hemodynamic instability, including prolonged capillary refill, mottled skin, hypotension, and tachycardia. Reported clinical features correspond to the presentation at the time clinical suspicion prompted blood culture collection. Respiratory support was required in all patients: non-invasive respiratory support (CPAP or non-invasive ventilation) in 13 patients (81.3%), invasive mechanical ventilation in two (12.5%), and supplemental oxygen therapy alone in one (6.3%). Respiratory support was initiated because of the infection episode in all cases, rather than preceding it. UVC dwell time ranged from two to nine days; catheters were removed promptly upon confirmation of a positive blood culture in all catheterized patients. No neonate had a history of prior surgery or exposure to invasive devices other than the UVC and respiratory support.

Laboratory findings

Leukocyte counts ranged from 1,840 to 38,600/µL (median, 10,900/µL); leukopenia was defined as <5,000/µL and leukocytosis as >30,000/µL. Thrombocytopenia (platelet count <150,000/µL) was frequent and isolated, without accompanying coagulopathy suggestive of disseminated intravascular coagulation; severe thrombocytopenia (<50,000/µL) was observed in six patients (37.5%). CRP levels ranged from 4.8 to 160 mg/L. Blood cultures were positive for *S. marcescens* in all 16 neonates. Repeat blood cultures were obtained approximately every 48 hours after the initial positive culture and continued until microbiological clearance was documented, although exact clearance dates were not systematically recorded for all patients. Umbilical catheter-tip cultures were obtained in all 15 catheterized neonates: six were positive for *S. marcescens*, and nine were negative. Formal central-line-associated bloodstream infection (CLABSI) criteria were not systematically applied. Lumbar puncture was not performed in this cohort, as patients were hemodynamically unstable and had isolated thrombocytopenia at presentation, both of which precluded the procedure; meningitis could therefore not be formally excluded.

Imaging findings

Cranial ultrasonography was normal in most patients. Five neonates (31.3%) had grade I intraventricular hemorrhage, and one neonate (Case 16) had grade II intraventricular hemorrhage; cranial ultrasonography in this patient was normal prior to day 6 of life, when the hemorrhage was first identified. This same patient (Case 16) experienced a second episode of *S. marcescens* bacteremia on day 30 of life and was subsequently diagnosed, on day 60 of life, with multiple contrast-enhancing lesions on MRI in the bilateral frontal and right parieto-occipital regions, consistent with multifocal brain abscess formation rather than infarction or hemorrhage.

Treatment and outcomes

All patients received empirical intravenous imipenem and amikacin at treatment initiation. Amikacin was discontinued 48 hours after the initial positive blood culture, given confirmed in vitro resistance, and replaced by gentamicin, to which all isolates were susceptible; imipenem was continued. If blood cultures remained positive or no clinical improvement was observed after a further 48 hours, therapy was escalated to either ciprofloxacin or piperacillin-tazobactam. Total antimicrobial duration was 10 days following documented blood-culture clearance. Definitive antimicrobial regimens were distributed as follows: piperacillin-tazobactam (four patients), ciprofloxacin (six patients), oral fosfomycin (four patients; 100 mg/kg/day in four divided doses for 10 days following culture clearance, administered because of clinical failure after several prior antimicrobial lines, with favorable outcomes in all four patients), and imipenem-gentamicin maintained as definitive therapy (two patients who responded adequately to this regimen). UVCs were removed promptly upon confirmation of a positive blood culture in all catheterized patients, consistent with the reported catheter dwell times (two to nine days). Length of hospital stay ranged from 12 to 90 days. The primary outcome was all-cause in-hospital mortality: nine neonates survived (56.3%), and seven died (43.8%).

## Discussion

In this case series, we describe 16 episodes of *S. marcescens* infection in a NICU, highlighting its clinical severity and association with invasive procedures. Our findings are consistent with the growing body of evidence identifying *S. marcescens* as an important healthcare-associated pathogen in vulnerable neonatal populations.

Epidemiology and frequent characteristics

Consistent with prior reports, prematurity, LBW, and invasive supportive care (umbilical catheters and mechanical ventilation) were frequent characteristics among affected infants [1,2,5]. As this case series lacked an uninfected comparator group, these findings should be interpreted as frequent exposures rather than independently established risk factors. Similar associations between neonatal *S. marcescens* sepsis and invasive devices, including central venous catheters and mechanical ventilation, have been reported in other neonatal cohorts [1,5].

The near-universal presence of umbilical venous catheters (UVCs) in our cohort (15/16 patients) is notable; however, UVC use is also common throughout our broader NICU population, and this finding alone cannot establish umbilical catheterization as a confirmed portal of entry. Catheter-tip cultures were obtained in all 15 catheterized neonates; six were positive for *S. marcescens*. However, formal CLABSI criteria and precise timing data were not available, precluding definitive attribution of catheter-related infection.

Microbiology, resistance, and pathogenesis

*S. marcescens* is adept at colonizing hospital reservoirs, including disinfectants, soaps, water systems, and medical devices [2,6]. Outbreak investigations frequently fail to isolate a definitive environmental source; cross-transmission via staff hands is often the presumed reservoir.

*S. marcescens* carries an intrinsic chromosomal ampC gene; however, current guidance considers *S. marcescens* less likely than classic moderate-risk organisms such as *Enterobacter cloacae* complex, *Klebsiella aerogenes*, or *Citrobacter freundii* to develop clinically significant AmpC overexpression during therapy. Treatment should generally be guided by susceptibility results, although high-burden infections or inadequate source control warrant additional caution [7].

The antibiotic susceptibility profile observed in our series - resistance to ampicillin, amoxicillin-clavulanate, cefoxitin, cefadroxil, tobramycin, and amikacin - reflects the intrinsic chromosomal resistance pattern typical of *S. marcescens* [8]. Our isolates remained susceptible in vitro to the third-generation cephalosporin cefotaxime, as well as to carbapenems and piperacillin-tazobactam, in agreement with previous reports [8,9]. Cefepime, a fourth-generation cephalosporin, was not tested, and no claim of susceptibility to this class can be made. The finding of in vitro cefotaxime susceptibility should be interpreted with caution in the context of severe neonatal bacteremia, uncertain source control in some patients, reliance on a single local laboratory’s susceptibility standards, and the absence of molecular resistance testing. In contrast, our isolates were resistant to amikacin and tobramycin while remaining susceptible to gentamicin; this pattern differs from several published series in which aminoglycosides generally retain good activity against *S. marcescens* and may reflect local antimicrobial selection pressure or regional differences in resistance mechanisms [8,10].

No phenotypic or molecular testing for extended-spectrum β-lactamases, carbapenemases, or aminoglycoside-resistance mechanisms was performed. For infections caused by extensively drug-resistant Gram-negative organisms with limited conventional options, emerging pediatric experience with agents such as cefiderocol may be relevant; however, neonatal evidence remains limited, and treatment should be individualized [11]. None of our isolates met criteria for extensive drug resistance, remaining susceptible to multiple antimicrobial classes.

Clinical and imaging findings

The clinical presentation in our cohort - non-specific signs of sepsis, respiratory distress, and hemodynamic instability - is typical of neonatal sepsis and often delays targeted diagnosis. Thrombocytopenia observed in this cohort was isolated, without accompanying coagulopathy suggestive of disseminated intravascular coagulation. Neurologic imaging seldom reveals findings in neonatal *S. marcescens* infection. Although rare, central nervous system involvement, including meningitis and brain abscess, has been reported in association with *S. marcescens* infection and is associated with poor prognosis [12,13]. In our series, one infant (Case 16) developed multiple contrast-enhancing lesions on MRI in the bilateral frontal and right parieto-occipital regions, consistent with multifocal brain abscess formation rather than infarction or hemorrhage. This patient experienced a second episode of *S. marcescens* bacteremia on day 30 of life, with abscesses diagnosed on day 60 of life; this case, including representative imaging, has been described in greater detail in a related report by our group [14]. Vigilance is warranted in neurologically symptomatic infants, and early neuroimaging should be considered even in the absence of classical meningeal signs.

Outcomes and mortality

Our observed mortality (43.8%) is high but within the range reported specifically for neonatal *S. marcescens* infection. Neonatal-specific mortality rates range from 7% in a 10-year NICU surveillance study [2] to 14.3% in an outbreak series [15], and up to 50% in another neonatal septicemia series, situating our findings within the upper range of this neonatal-specific literature. Poor outcomes in our cohort were observed predominantly among preterm and low-birth-weight neonates; however, mortality among neonates weighing <1,500 g (4/9) was not markedly different from that among neonates weighing ≥1,500 g (3/7), and this study was not designed to formally test this association. All-cause in-hospital mortality is reported here; infection-attributable mortality could not be reliably determined. The small sample size in this series may amplify fluctuations in fatality estimates.

Prevention and infection control

Effective infection control in NICUs is essential given the frequent association between *S. marcescens* and invasive procedures such as catheterization and mechanical ventilation. Rigorous hand hygiene remains the cornerstone of prevention, supported by strict aseptic technique during catheter insertion and maintenance and early removal when no longer required [2,6]. Environmental cleaning, cohorting or isolation of infected neonates, and staff education are additional recommended measures [2,6]. Stool or rectal screening of healthcare staff and co-hospitalized neonates was not performed during our study period, as this was not conducted as a formal outbreak investigation; such screening is recommended in future investigations when temporal clustering of *S. marcescens* cases is suspected, given the recognized role of gastrointestinal colonization as a potential reservoir. Molecular typing methods, including whole-genome sequencing, facilitate identification of transmission routes and source tracking when clustering is suspected [16].

Limitations

This study has several limitations. It is a retrospective, single-center case series with a small sample size, which limits generalizability and precludes multivariate analysis. The absence of an uninfected comparator group means that prematurity, LBW, umbilical catheterization, and respiratory support should be regarded as frequent characteristics of the cohort rather than independently established risk factors. The 4.2% proportion of admissions affected should not be interpreted as an incidence density, as patient-day and central-line-day denominators were not available and baseline *S. marcescens* colonization or infection rates in our unit were not systematically documented. No molecular typing or environmental/colonization surveillance was performed, precluding confirmation of clonal transmission despite the identical antibiogram observed across isolates. Catheter-tip culture positivity could not be formally linked to catheter-related bloodstream infection, as CLABSI criteria and precise catheter-removal timing relative to blood-culture positivity were not systematically documented. Antimicrobial dosing details were available at the regimen level but not individually verified for every patient, and repeat blood-culture clearance dates were not systematically recorded. The interpretation of fosfomycin susceptibility is limited by the absence of a CLSI-validated breakpoint for *S. marcescens*. All-cause in-hospital mortality was reported, and infection-attributable mortality could not be reliably determined. The exclusion of neonates with major congenital malformations or congenital heart disease, while intended to avoid confounding attribution of death, may have introduced selection bias by removing a high-risk subgroup. Finally, post-discharge neurological follow-up data were not available, limiting assessment of long-term outcomes.

## Conclusions

This single-center retrospective case series documents severe blood-culture-confirmed *S. marcescens* infection among hospitalized neonates, with seven deaths among 16 affected patients. Prematurity, LBW, UVC exposure, and respiratory support were common characteristics, although the absence of a comparison group prevents identification of independent risk factors. Timely microbiological diagnosis, susceptibility-guided treatment, central-line care, and structured infection-prevention surveillance are essential. When temporal clustering is suspected, detailed epidemiological investigation and molecular typing should be pursued.
